# Pitfalls using smartphones videos in diagnosing functional seizures

**DOI:** 10.1016/j.ebr.2021.100497

**Published:** 2021-11-08

**Authors:** Brin Freund, William O. Tatum

**Affiliations:** Department of Neurology, Mayo Clinic, 4500 San Pablo Road, Jacksonville, FL 32224, United States

**Keywords:** Smartphone, Video, Seizures, Post-ictal, Misdiagnosis

## Abstract

Expert review of seizure semiology looking at video recordings independent of EEG has been found to be useful for diagnosing functional seizures. Videos recorded outside the hospital containing “spells” have similar sensitivity to EEG when quality recordings are evaluated. Recently, smartphone videos were shown to serve as an adjunct to standard history and physical examination with similar diagnostic yields when compared to diagnostic video-EEG monitoring and reviewed by experts. However, caution must be exercised when interpreting videos of paroxysmal neurological events recorded by caregivers to ensure proper video quality is maintained and recorded event is representative. In this report, we present a case of initial identification of and event falsely suggesting functional seizures in a patient with epilepsy. The smartphone video of a “seizure” was recorded by his wife using her smartphone. Despite a quality recording and a history consistent with epilepsy, the smartphone video reviewed during evaluation in the clinic suggested a functional behavior in contrast to the history that suggested epilepsy manifest as convulsions. Instead of bilateral tonic-clonic motor movements, bizarre, intermittent non-clonic wild flinging movements and vocalization were identified on the smartphone video. The discordance between the clnical history and ideo prompted inpatient video-EEG monitoring. The same nonepileptic semiology was subsequently clarified to represent a physiological nonepileptic event. The event on the smartphone was typical of his agitated post-ictal state following an electroclinical tonic-clonic seizure. With treatment the seizures became controlled with antiseizure medication in long-term follow-up. We highlight the pitfalls using patient-recorded smartphone videos in patients diagnosed with epilepsy. Understanding the utility of smartphones as an adjunct to the clinical history will help in differentiating epileptic from functional seizures.

## Highlights

• Smartphone videos are useful cost-effective means to evaluate and characterized epileptic and nonepileptic seizures.
• Convulsive and motor semiologies are particularly well-suited for accurate interpretation using smartphone videos but require ictal-post-ictal separation.
• Patients and caregivers should be educated on optimal smartphone video recording focused on capturing the onset of the event.
• Long-term video-EEG monitoring remains the gold standard when discordant video information is presented or diagnostic questions remain after clinical evaluation.

## 1 Introduction

Seizures are one of the most common reasons for patients to consult with neurologists [1]. Diagnosis and classification of seizures, including differentiating epileptic seizures from functional seizures (FS), is often inferred from descriptions provided by patients and witnesses of the events [2]. However, identifying and classifying seizures can be difficult using bystander report alone [2], [3], [4], [5]. Misdiagnosis is common [3] when relying on descriptions of the events as opposed to direct observation by a specialist with experience treating seizure disorders [2]. Inpatient long-term video-EEG monitoring (LTVEM) is the gold standard for establishing a definitive diagnosis of FS when the typical event is captured and both the semiology and concurrent EEG are consistent [3], [6], [7], [8]. Still multiple barriers to inpatient LTVEM exist including significant costs, accessibility, availability, and insurance coverage that require intensive resource utilization [9]. Therefore, LTVEM and referral to a full-service epilepsy center early in the course of the assessment is advised when the diagnosis remains in question [3], [8], [10], [11], [12]. In addition, there is an inherent unpredictability of event capture and the duration of LTVEM may not be sufficient to capture the event in question [13], [14]. Ambulatory video-EEG monitoring [15], [16] is more readily available on shorter notice than LTVEM but has other limitations including short duration as well as limitations of drug-reduction and on-site intervention to assess the patient during recording that is possible with inpatient evaluation.

The value of clinical semiology in diagnosing epileptic seizures has been demonstrated [17], [18]. Providing an early diagnosis of FS as one that is separate from epileptic seizures is crucial to ensure appropriate treatment [2], [19], [20], [21]. However, unlike portable video recording, EEG monitoring may not be readily available. Therefore, alternative techniques from LTVEM are required to facilitate an accurate diagnosis. With this is in mind, home video recordings have been evaluated and have been shown to be useful in diagnosing FS [22].

**Fig. 1:**
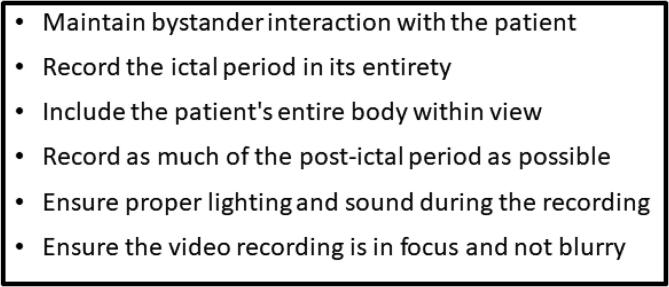
Recommendations for quality smartphone video recording during seizure. Adapted from Tatum, et al., 2021 [26].

Smartphones are ubiquitous [5], [23], easy to use, and are available immediately for recording any events that may arise at a given time. One study of a diverse population in the US showed 96% of people surveyed had access to a mobile phone and 83% own a smartphone [24]. On the other hand, there are only 230 accredited specialized epilepsy centers in the US where patients can be admitted and monitored using video-EEG [25]. Smartphones are capable of providing high quality video [26] that can be utilized to record FS and other events [3]. However, smartphone videos are not recorded in a controlled environment in which the clinicians themselves are able to define when and how each spell is to be recorded, as with continuously recorded video-EEG performed with either inpatient or outpatient EEG monitoring. As such, there are issues of focus, lighting, duration, and initiation that must be considered when interpreting homemade smartphone video recordings obtained by a lay population of caregivers to evaluate people with seizures [26] (Fig. 1).

In this report, we illustrate the pitfalls in using video recordings obtained by patients and family members with their smartphones when used as an adjunct to evaluate people with seizures to avoid confusion and misdiagnosis.

## 2 Case report

A 25-year-old man presented to our epilepsy center for evaluation of seizures. He was born at term without any developmental delays and had no risk factors for epilepsy including traumatic brain injury, brain surgery, febrile seizures, central nervous system infections, or family history of seizures and no significant past medical or psychiatric comorbidities. Three years prior to his presentation he had his first seizure. He did not remember the event, but while attending basic training in the Army, he was reportedly found in the shower confused by his fellow soldiers. There was no tongue bite or urinary incontinence, but he was disoriented afterward for much of that day. He had another episode within the same month while he was performing physical training exercises, whereby he collapsed and remained confused for hours, but no report of witnessed convulsions. An evaluation at that time was unrevealing. He had 12 episodes in the next 3 years. They were all similar, some associated with lateral tongue laceration suffered during the event. He was seizure-free for 6 months and then began to have spells at least monthly. He denied an aura or premonition preceding his seizures. His wife reported at night that he would “cry” at the onset and then appears to have clonic jerking bilaterally and symmetrically, up to 3 minutes in duration. He was reported to be distressed for a few minutes after the episodes. Brain MRI was reportedly normal and EEG abnormal, but the reports were unavailable. He had been taking levetiracetam 3000 mg daily with topiramate 50 mg daily. He had also tried valproic acid but reportedly had abnormal labatory studies so this was discontinued. At his appointment, it was determined that he would continue his current regimen of levetiracetam, and topiramate was increased to 100 mg total daily. A presumptive diagnosis of epilepsy was made upon clinical grounds though the classification included focal epilepsy localized to the frontal head region or genetic generalized epilepsy manifest as recurrent nocturnal generalized tonic-clonic seizures. At his follow up appointment, a high-resolution 3-T brain MRI was performed and was normal without intracranial abnormalities. EEG demonstrated 3–4 Hz generalized polyspike-and-wave discharges supporting a clinical diagnosis of genetic generalized epilepsy. The patient and his wife had recorded a video of his habitual seizures, which was reviewed an epileptologists (WOT). As noted in the video, he appears agitated and combative and is thrashing his extremities in a non-rhythmic and discontinuous manner with side to side head movements with eyes closed (Video 1). He and his wife were clear that this was the semiology of his typical seizure. The side to side head movements, eye closure, and discontinuous nonrhythmic hypermotor activity suggested FS [18]. He was subsequently admitted to the epilepsy monitoring unit for LTVEM for differential diagnosis and classification of recurrent events. During the admission, EEG redemonstrated interictal generalized spike and polyspike and slow wave complexes noted previously. He had one seizure with clinical semiology suggesting a focal to bilateral tonic-clonic seizure due to head version, yet lateralized and focal seizures are known to occur in genetic generalized epilepsies [27]. Despite the appearance of focal features, the ictal EEG demonstrated a generalized seizure onset. Immediately following a definitive diagnosis of epilepsy with electroclinical support from a electroclinical bilateral tonic-clonic seizure, he exhibited the exact same post-ictal behavior that was witnessed in clinic while reviewing the smartphone video. This behavior observed on the smartphone video was therefore able to be linked to his habitual postictal state with violent thrashing that simulated a FS (Video 2). In discussion with the patient and his wife, the difference between his seizure and a postictal state with confusion and combativeness was underscored to define a sequence of events rather than separate events. LTVEM was therefore able to establish a diagnosis of genetic genealized epilepsy despite the history suggesting focal epilepsy and the smartphone video suggesting a FS.

## 3 Discussion

This case highlights the pitfalls of using patient-recorded smartphone videos alone as a diagnostic tool without considering history, physical examination, and ancillary testing. Despite the patient correctly diagnosed with epilepsy on clinical grounds, classification remained unclear and the smartphone video served in this case to be a pitfall in deference to the history based upon a post-ictal semiology that was recorded supporting a nonepileptic event. This subsequently led to an otherwise potentially unavoidable LTVEM session where discordant information could be resolved by other means and provide a definitive diagnosis or establish a dual diagnosis.

Recent studies have demonstrated the utility of smartphone videos in evaluating patients with FS when a high-quality ictal recording is obtained, especially when convulsive activity is present [3], [5], [23]. When videos are used in conjunction with history and physical examination, these video-based diagnoses have been found to be highly specific as well as sensitive when compared to the gold-standard inpatient LTVEM [3]. Video-based diagnosis using a smartphone can be especially useful in establishing a LTVEM diagnosis of FS; in one study, 25% of videos of FS showed 100% concordance with LTVEM supported diagnosis [23]. The diagnosis of FS is particularly accurate when the event in question demonstrates convulsive motor activity [3]. This is consistent with a previous study of inpatient video recordings [28]. However, it is vital that these recordings are reviewed by a neurologist with expertise treating patients with epilepsy to differentiate epileptic seizures from FS based upon video recording alone. This has limitations for medical professionals who lack epilepsy training [3], [24] and for non-neurologists [29].

As opposed to inpatient LTVEM [2] patient-derived smartphone videos alone may lead to false negative diagnoses as in the present case where our patient had a confirmed diagnosis of epilepsy. The patient and family in this case lacked expertise and intrinsic information which lead to failure to record the onset of the seizure. By virtue of the mechanics of video recording, loss of seizure-onset is an expectation. This is at odds with continuous LTVEM that is performed in the hospital with continuous video-EEG monitoring during event recording [3]. Further, it has been demonstrated that laypeople as bystanders have difficulty discriminating between epileptic and nonepileptic events [4]. It stands to reason they may have difficulty discerning ictal from postictal activity. These issues may affect their historical and linguistic ability to reliably represent the event in question. Therefore, one must be careful when using video recording provided by the patient and/or family and ensure that what the patient and the camera operator have filmed is the “ictal” activity in question and not the post-ictal state or an atypical event separte from the habitually recurring episodes.

This case further highlights the importance of separating and defining ictal and postictal periods. The recorded post-ictal state felt to reflect an “ictal” event was prolonged and involved violent and disorganized movements of the extremities, both features that suggested FS [30] especially because ictal violence is rare and often very brief [31]. On the other hand, if viewed as a postictal recording, epilepsy would be a more likely explanation than a FS as the postictal state tends to have a longer duration of confusion prior to return to baseline in people with epileptic seizures [30]. Patients with a prolonged convulsive seizure are subject to postictal delirium. This can involve agitation and combativeness lasting up to 1–2 days in some cases [32]. In this case, his hyperactive delirium due to a postictal state manifest as violent behavior is much more typical of a postictal state as opposed to being due to an ictal phenomenon [33]. Postictal states are most commonly associated with a reduced level of responsiveness compared with agitation or psychosis [34]. This makes interpretation of the video even more difficult when taking this into account.

Therefore, defining guidelines for the use of standardized outpatient smartphone video recordings to be used for clinical diagnosis is key when educating the patient and family to ensure optimal information is available for the clinician. In this case, the history that was obtained was consistent with a diagnosis of epilepsy blurred between focal and generalized epilepsies, however, the video suggested nonepileptic event potenetially reflecting a FS. Proper instruction and education of patients and their families regarding acquiring smartphone videos can help streamline resource utilization and potentially minimize healthcare expenditures by avoiding unnecessary testing. Clinicians who interpret the semiology observed in smartphone video should specifically inquire about the subjective symptoms and semiology before and after the time documented by the video to establish the full duration and context of the event that is viewed.

Smartphone videos are a time-efficient adjunctive tool that provides complementary information to the standard history and physical examination [3]. The atypical post-ictal state represented by the smartphone video was unique and in contrast to the clinical history as opposed to not providing complementary information. Therefore, we feel it is important to outline the noise level that may occur when evaluating smartphone videos when evaluating patients to arrive at a clinical diagnosis based on history and physical examination, so misdiagnosis and excessive testing is avoided. When discordance exists between the clinical history and physical examination and smartphone video review, inpatient LTVEM should be pursued to establish a definite diagnosis and ensure proper treatment of patients.

## 4 Conclusion

Smartphone video recordings of seizures and seizure mimics are an increasing source of supplemental information provided by patients, family, friends, and caregivers to clinicians. These smartphone videos are useful as a clinical adjunct in concert with standard history and physical examination to arrive at a diagnosis in patients with paroxysmal neurological events. Despite the high sensitivity and specificity of high quality videos when viewed by experts, pitfalls may arise when over-emphasis is placed on videos alone as illustrated by this case. Patients and caregivers should be educated on when, what, and how to record a smartphone video at home during the typical patient event. Clarifying the importance of capturing the “ictal” state as close to the onset as possible while video recoding the entire event is crucial for ensuring accuracy. When discordant information from a smartphone video is submitted or when in doubt of the diagnosis, inpatient LTVEM should be pursued to obtain a definitive diagnosis. Smartphone videos will continue to increase in use as mobile health advances in many areas of medicine where functional neurological disorders exist.

## Ethical statement

A signed informed consent was obtained from the patient to present their video information to be used in this publication as a separate consent from that obtained for video-EEG monitoring.

## Declaration of Competing Interest

The authors declare that they have no known competing financial interests or personal relationships that could have appeared to influence the work reported in this paper.

## Appendix A Supplementary data

Supplementary video 1 Outpatient smartphone video demonstrating an unresponsive patient with eyes closed, non-rhythmic flailing arm movements, and side to side head movements which appeared consistent with a functional seizure.

Supplementary video 2 Electroclinical focal to bilateral tonic-clonic seizure with postictal agitation captured during inpatient video-EEG monitoring. The semiology of the postictal state was identical to that presented on the outpatient smartphone video 1 viewed in clinic which confirmed the video provided from the smartphone recording was the patient’s postictal agitation, and not his habitual seizure.
